# A Mixed Methods Case Study on Extreme Weather Preparedness Among Older Adults in Low-Income Housing

**DOI:** 10.1093/geroni/igaf122.1431

**Published:** 2025-12-31

**Authors:** Holly Dabelko-Schoeny, Smitha Rao, Anthony Traver, Josie Sarabia, Emma Rademacher, Max Stokey, Marisa Sheldon

**Affiliations:** The Ohio State University, Columbus, Ohio, United States; Ohio University, Columbus, Ohio, United States; The Ohio State University, Columbus, Ohio, United States; The Ohio State University, Columbus, Ohio, United States; The Ohio State University College of Public Health, Columbus, Ohio, United States; The Ohio State University, Columbus, Ohio, United States; The Ohio State University, Columbus, Ohio, United States

## Abstract

Disasters and extreme weather events are no longer considered rare; they routinely disrupt daily routines and challenge people’s ability to maintain productivity and health. Older adults and individuals with disabilities living in low-income housing often bear the brunt of these occurrences, including extreme heat, cold, winter storms, hurricanes, and excessive precipitation. Using the Aging in the Right Place (AIRP) and cumulative disadvantage frameworks, this convergent mixed methods case study examined older adults’ experiences with extreme weather, focusing on preparedness and response in low-income housing. Data from survey responses (N = 40) and focus group discussions (N = 26) were analyzed using constant-comparison analysis. Residents’ understanding of extreme weather preparedness and response was mapped onto elements of the disaster risk management cycle, informed by the theory of planned behavior. Despite differing views on extreme weather risks, residents considered preparedness vital for safety, especially for isolated or disabled individuals. The built environment, information sources, and social connections influenced attitudes and actions related to extreme weather. Based on study findings, a toolkit was developed for use by human services professionals working in low-income affordable housing properties, including identification of resident risk, resident-informed informational flyers, and space assessment worksheets. This study supports the co-development of community-informed regional disaster preparedness plans, particularly for individuals in affordable housing communities.

